# Does Preoperative Weight Loss Predict Significant Postoperative Weight Loss Among Patients who Underwent Laparoscopic Sleeve Gastrectomy?

**DOI:** 10.7759/cureus.5870

**Published:** 2019-10-09

**Authors:** Ugochukwu Chinaka, Joshua Fultang, Abdulmajid Ali

**Affiliations:** 1 General Surgery, University Hospital Ayr/University of West of Scotland, Ayr, GBR; 2 General Surgery, University Hospital Ayr/University of West of Scotland, Ayr, GBR

**Keywords:** preoperative weight loss, bariatric surgery, sleeve gastrectomy, target preoperative weight loss, postoperative weight loss

## Abstract

Background

Some bariatric practices, mainly those conducted under public-funded services, have adopted achieving a target preoperative weight loss (PrWL) before offering surgery. There are varied opinions on the correlation between preoperative and postoperative weight loss (PoWL) for the different surgical options. This study investigated the impact of target PrWL on PoWL for patients who underwent laparoscopic sleeve gastrectomy (LSG).

Materials and methods

A longitudinal retrospective cohort study was carried out on patients who had documented preoperative weight before LSG (n=155) from the authors’ institution between February 2008 to October 2017. Patients were grouped into two cohorts based on meeting the 5% target PrWL or not. The endpoint included percent postoperative weight loss (% PoWL) at one year and two to three years.

Results

A total of 155 individuals were identified and analysed. Of these patients, 78.7% of them (n=122) achieved the 5% target PrWL (target group) while 21.3% (n= 33) did not (non-target group). At one year, there was no statistical significant difference in the mean % PoWL between the non-target and target groups (22.3 ± 8.1% versus 19.4 ± 11.8% p value= 0.08). A similar observation was made at two-three years, where the mean % PoWL in the non-target group was 14.7 ± 10.7% versus 16.3 ± 14.4% in the target group (p value= 0.07). Our further analysis highlighted a statistically significant weak inversely proportional correlation between % PrWL and % PoWL at one year and two to three years.

Conclusion

Meeting target PrWL does not significantly impact on PoWL after LSG. Therefore, it should not serve as exclusion criteria for eligible patients who are in need of surgery.

## Introduction

The stark epidemic proportion of obesity is a reality in today’s world [1]. The World Health Organization (WHO) in 2014 revealed estimates that show a staggering 39% of adults to be overweight and 13% obese [2]. Obesity is associated with comorbidities such as metabolic disease (such as type 2 diabetes), cardiovascular and joint disease, certain types of cancer, reduction in self-reported quality of life, and increased mortality in the long run [3]. It poses a huge health burden on the individual and has a large societal strain in terms of costs associated with human resources, administration, and long-term patient management [4].

Surgical intervention has been shown to result in greater improvement in terms of weight loss and obesity-associated comorbidities when compared with non-surgical interventions, regardless of the procedure [5]. This epidemic proportion of obesity has witnessed an attendant increase in laparoscopic bariatric surgery worldwide with estimates of about 468,609 procedures performed as of 2013. The most significant rise was that of laparoscopic sleeve gastrectomy (LSG) from 0 to 37% of the world total from 2003 to 2013 [6].

The criteria set out by the National Institutes of Health (NIH) Consensus Development Conference panel for patients requiring gastric restriction or bypass procedures include well-informed and motivated patients with acceptable operative risks, body mass index (BMI) exceeding 40 or between 35 and 40 with comorbidities (such as severe sleep apnoea, diabetes mellitus) and obesity-induced physical problems. It does not stipulate mandatory target preoperative weight loss (PrWL) [7].

Target PrWL is often encouraged amongst bariatric practitioners mainly public service funded services, before undergoing bariatric surgery to improve patient compliance and outcomes [8-10]. However, the impact of PrWL on a postoperative outcome such as postoperative weight loss (PoWL) has remained controversial [11]. Some have challenged the requirement and efficacy of a preoperative target weight loss before undergoing bariatric procedure [12].

Therefore, there is still a need to further investigate the significance of PrWL on PoWL, especially in patients who underwent LSG.

## Materials and methods

This study utilised de-identified data from our bariatric database of patients at the authors’ institution.

Cohort selection

We identified all patients who underwent any bariatric operation (gastric band, Roux-en-Y gastric bypass, sleeve gastrectomy, or revisional surgery) (n=339) from February 2008 to October 2017. A total of 166 LSG were performed (included 11 revisions from gastric bands to LSG); however, follow up data for 155 patients were obtained and those without documented postoperative weight after one year excluded from the analysis. Patients were classified as “target” and “non-target” categories based on meeting the 5% target PrWL or not, and the two groups were compared.

Variables

We focused on two variables. Percent PrWL (% PrWL) was determined by subtracting the weight at surgery from the recorded weight at referral to the bariatric service (initial weight) divided by initial weight multiplied by 100. Percent PoWL (% PoWL) was defined as the difference of the post-surgery weight at one, or two-three years from weight at surgery over the initial weight and expressed as a percentage.

Outcome measure

Our primary outcome was % PoWL at one-year and two-three years follow-up. At our institution, we follow up postoperative weights three months to 24 months postoperatively.

The Pearson correlation coefficient (r) was used to measure the relationship between both variables. F-test was used to determine the overall statistical significance of this relationship. All data analysis was performed with Microsoft Excel 2013.

## Results

**Figure 1:**
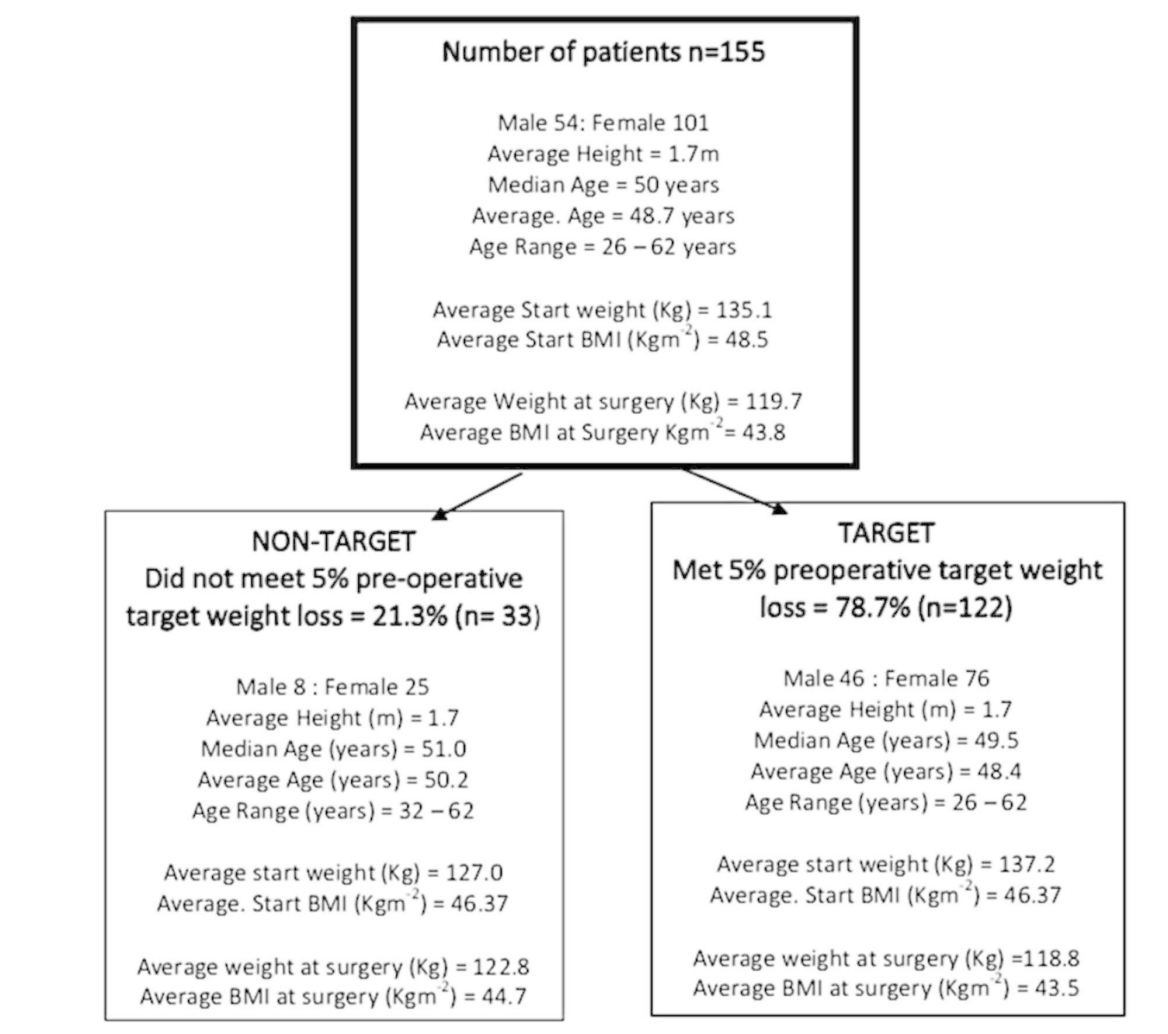
Number of patients and division into two cohorts based on meeting/not meeting the 5% PrWL target Both cohorts were comparable in terms of weight and body mass index (BMI) at referral and surgery. The upper limit of the subject's age was similar but the youngest patient in the cohort who did not meet the 5% PrWL target was 32-years-old, as compared to the 26-years old-in the other cohort. PrWL: preoperative weight loss.

Data from 155 patients was analysed. The median age was 50 years and there were more female than male patients. The average BMI at referral to the bariatric services (start BMI) was 48.5 Kgm^-2^. The average BMI at surgery was 43.8 Kgm^-2^ reflecting an average net weight loss preoperatively from an average of 135.1 Kg to 119.7 Kg at surgery. As seen in Figure 1, of the 155 patients, 78.7% of patients (n=122) achieved the 5% PrWL (target) while 21.3% (n=33) did not (non-target). The youngest subject was 26 years and the oldest 62 years at the time of surgery. 

**Figure 2:**
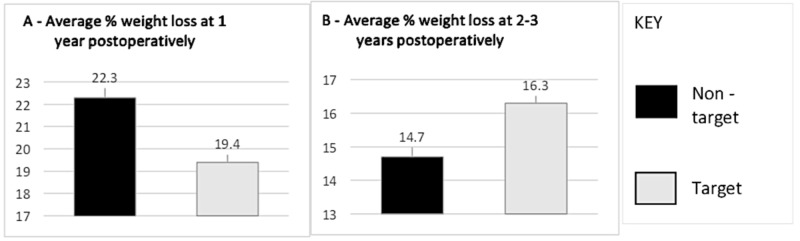
Average percentage of weight loss at one year (A) and two to three years (B) postoperatively At one year postoperatively, non-target group % PoWL was 22.3 ±8.1% (n=92) vs target 19.4 ±11.8% (n=22) (p value= 0.08). At two to three years postoperatively, non-target % PoWL was 14.7 ±10.7% (n=55) vs target 16.3 ±14.4% (n=21) (p value= 0.07). PoWL: postoperative weight loss.

At one year, patients who didn’t meet 5% target PrWL had an average % PoWL of 22.3 ±8.1% versus 19.4 ±11.8% for those who met 5% target PrWL (p value = 0.08) (Figure 2A).

At two to three years, the average % PoWL for patients who did not meet the 5% target PrWL was 14.7 ±10.7% (Figure 2B). This was slightly lower than the average % PoWL (16.3 ±14.4%) recorded in the cohort of subjects who met the 5% PrWL target (p value =0.07).

**Figure 3:**
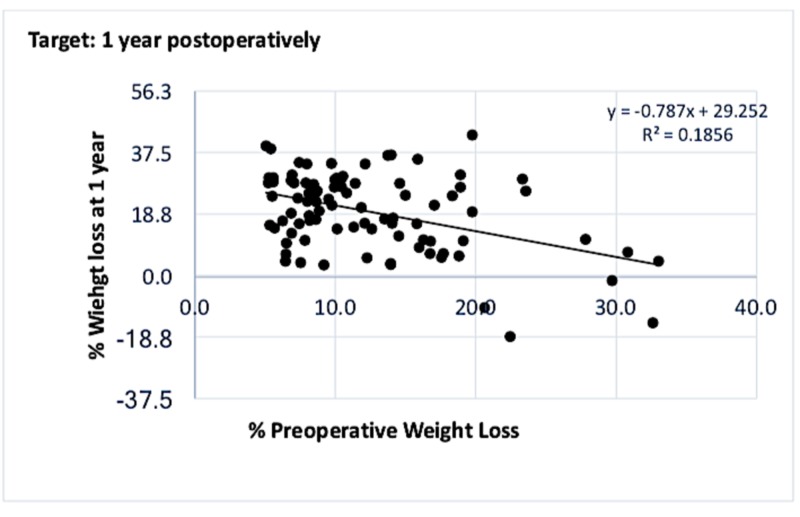
Graph showing correlation between % PrWL and % PoWL at one year in the cohort who met the 5% preoperative weight loss target Weak inversely proportional relationship in the target group at one year postoperatively. PrWL: preoperative weight loss; PoWL: postoperative weight loss.

**Figure 4:**
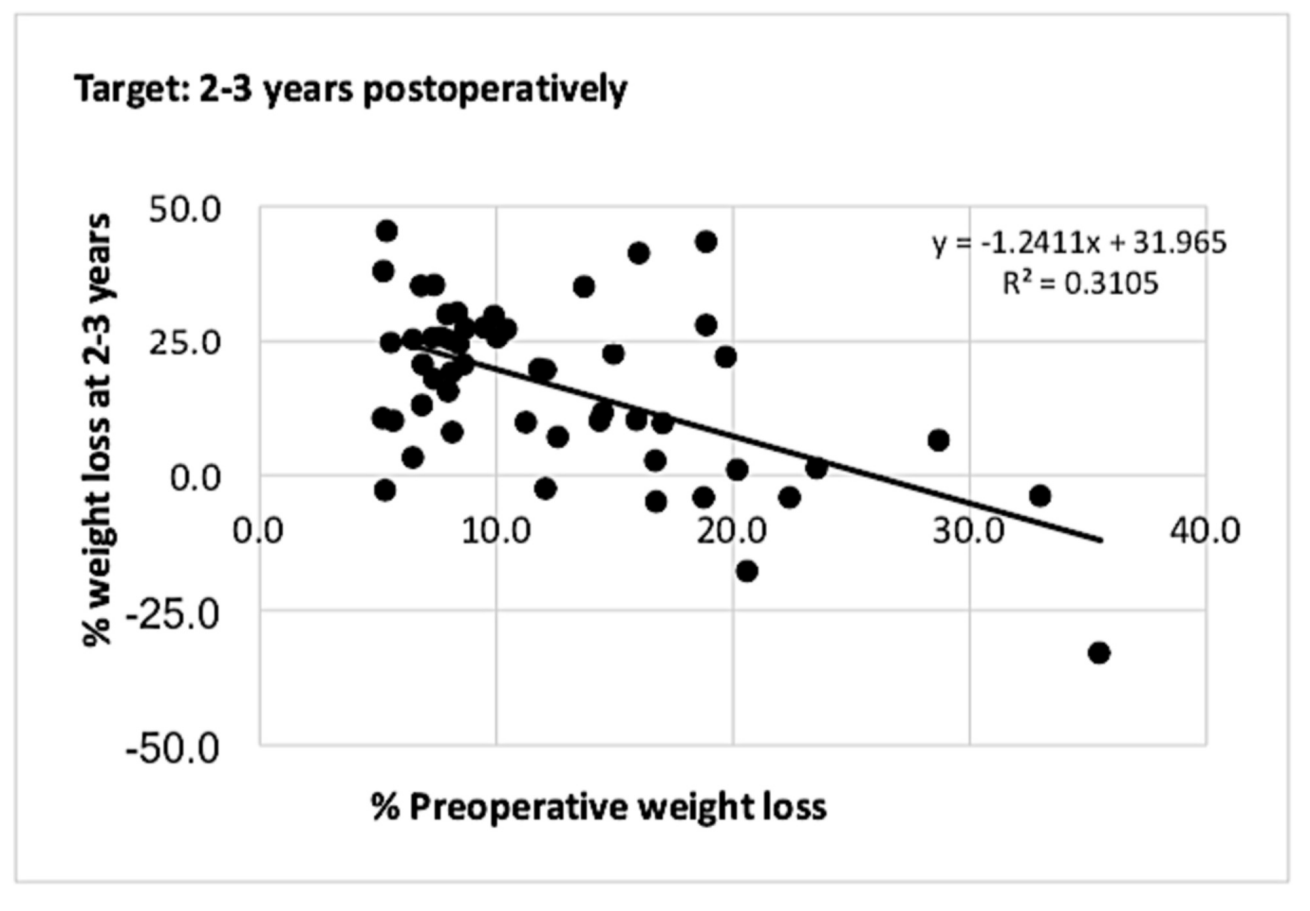
Graph showing correlation between % PrWL and % PoWL at two to three years for patients who met the 5% preoperative weight loss target At two to three years, there was a maintained weak inversely proportional relationship between % PrWL and % PoWL. PrWL: preoperative weight loss; PoWL: postoperative weight loss.

In the target group, the correlation between PrWL and PoWL at one and two to three years are detailed in Figures 3-4.

**Figure 5:**
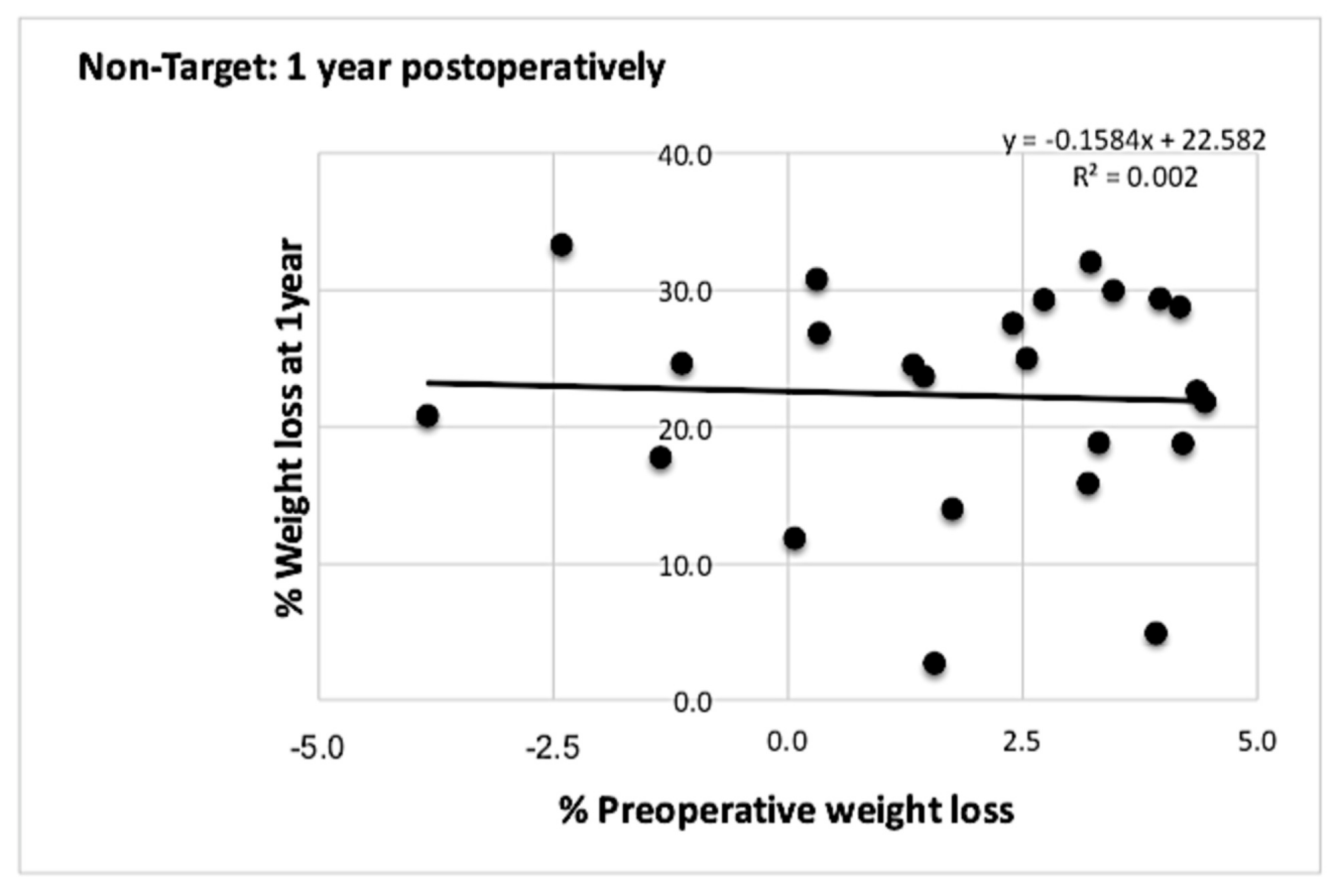
Graph showing correlation between % PrWL and % PoWL at one year for patients who did not meet the 5% preoperative weight loss target There was no correlation between % PrWL and % PoWL at one year postoperatively with r values closer to zero. PrWL: preoperative weight loss; PoWL: postoperative weight loss.

**Figure 6:**
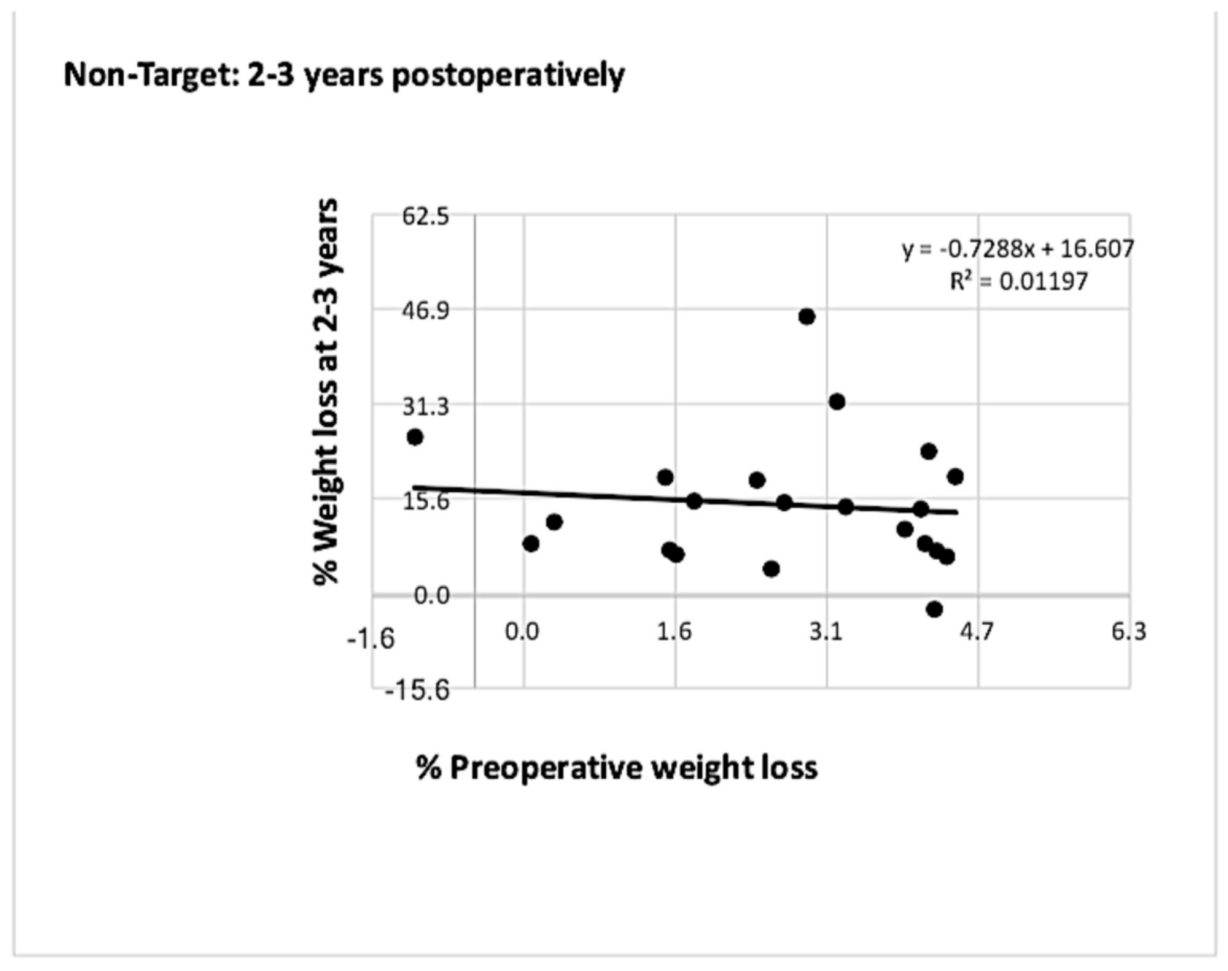
Graph showing correlation between % PrWL and % PoWL at two to three years for patients who did not meet the 5% preoperative weight loss target The correlation observed in the non-target group at one year was maintained at two to three years. PrWL: preoperative weight loss; PoWL: postoperative weight loss.

In the non-target group of patients, a similar relationship between both parameters was observed (Figures 5-6).

**Figure 7:**
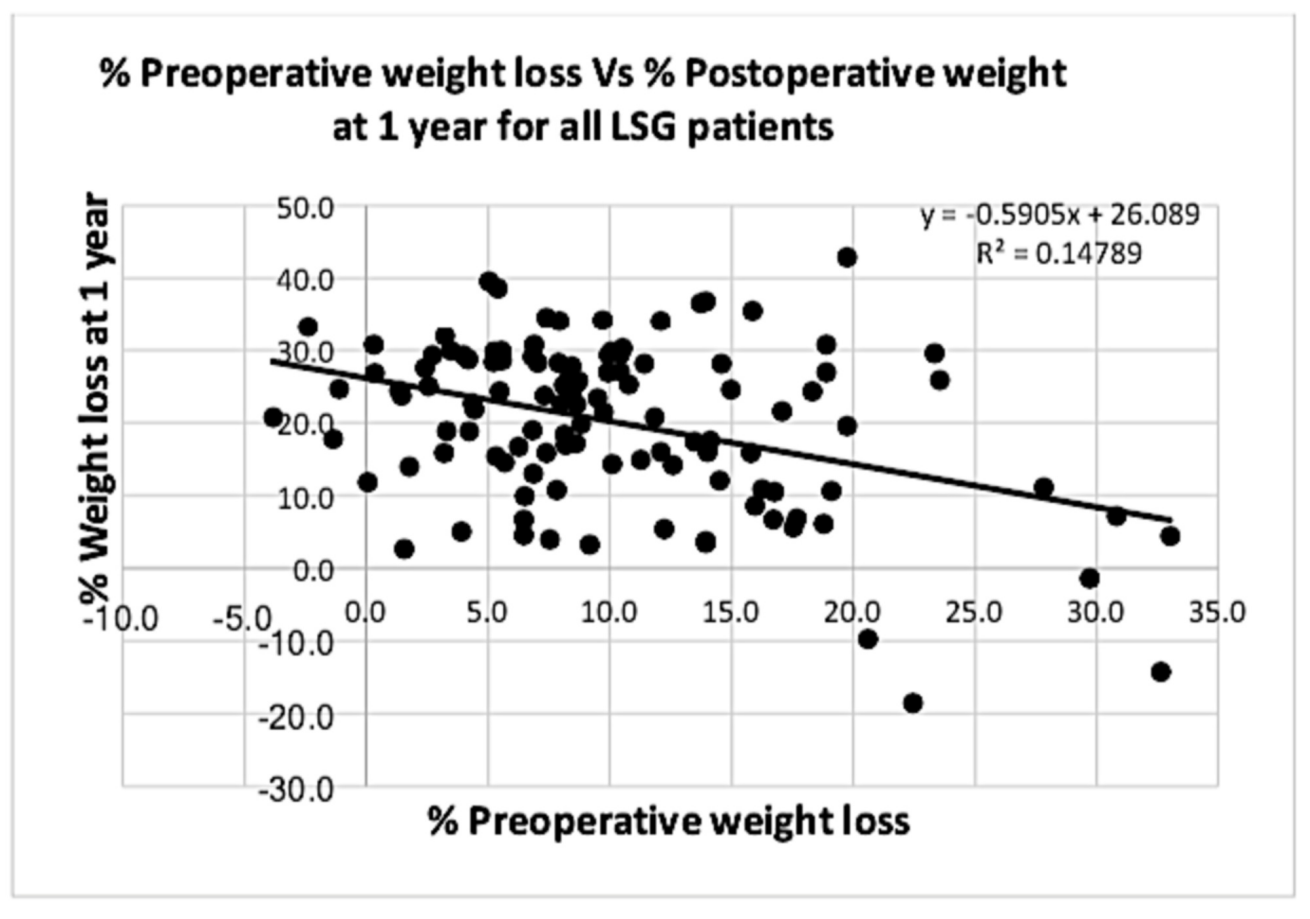
Graph showing correlation between % PrWL and % PoWL at one year for all laparoscopic sleeve gastrectomy (LSG) patients Analysing the correlation between % PrWL and % PoWL for all patients involved in the study showed a significant weak inversely proportional relationship at one year postoperatively (p value < 0.001). PrWL: preoperative weight loss; PoWL: postoperative weight loss.

**Figure 8:**
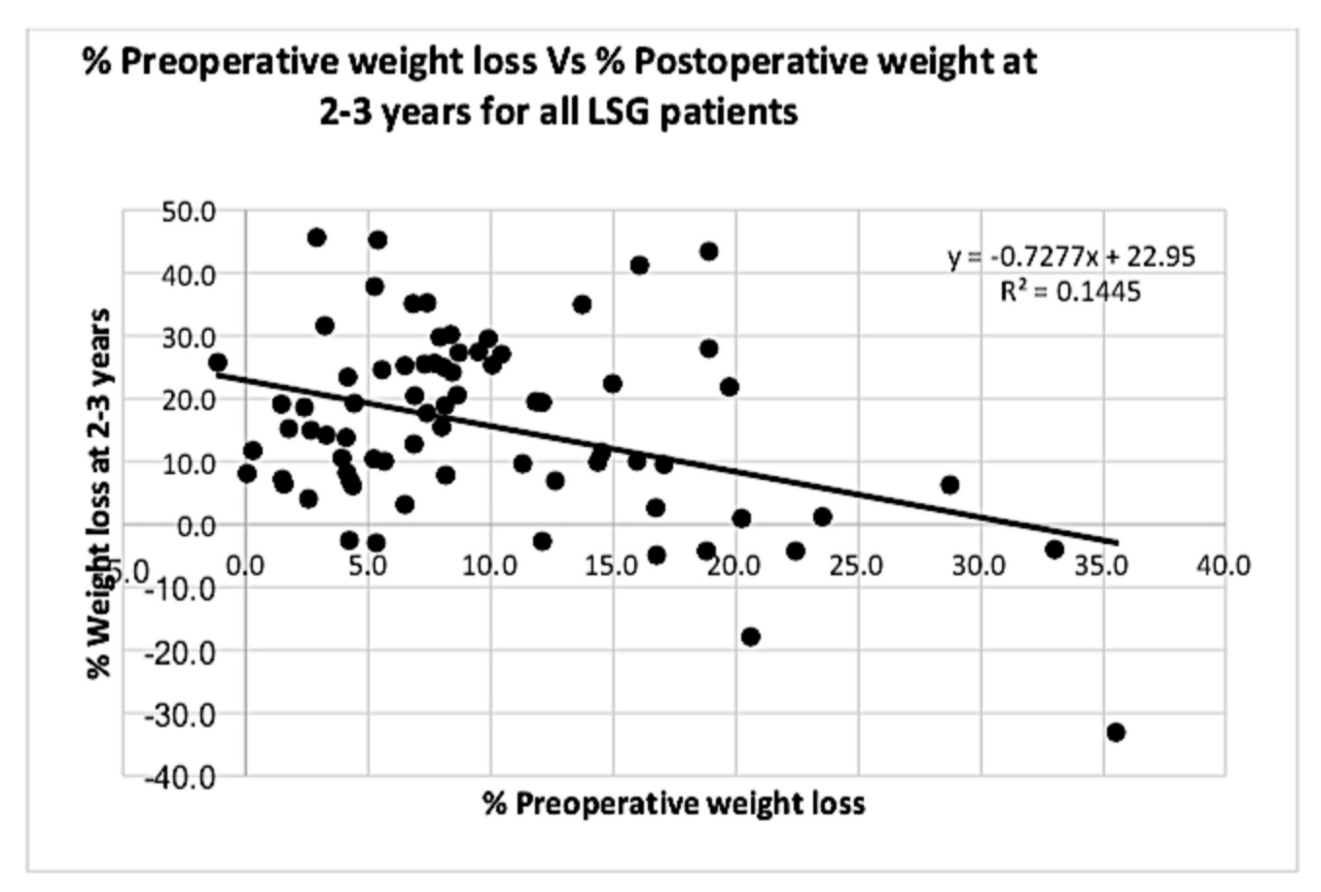
Graph showing correlation between % PrWL and % PoWL at two to three years for all laparoscopic sleeve gastrectomy (LSG) patients This statistically weak inversely proportional relationship was maintained at two to three years postoperatively (p value <0.001). PrWL: preoperative weight loss; PoWL: postoperative weight loss.

Analysing combined data from all patients who underwent an LSG is depicted in Figures 7-8.

## Discussion

Bariatric (weight loss) surgery as at today is safe, effective in producing significant sustainable weight loss, and leads to the improvement or resolution of co-morbidities associated with obesity [13-14]. LSG as a bariatric option has rapidly gained popularity and is considered the second most performed bariatric procedure in the world [15]. Despite the increased acceptance of weight loss surgery, less than 1% of eligible candidates end up receiving surgery in some climes [16]. In undertaking bariatric services, some institutions have adopted a policy of target PrWL citing advantages such as reduced peri-operative morbidity, decreased operating time with less blood loss, and possible motivation for further weight loss [17].

At the authors’ institution, the majority of the patients participated in the hospital’s preoperative program and are expected to achieve a target weight loss of 5% before undergoing surgery. However, some who did not meet the target weight loss but reasonably fulfilled the NIH Consensus Development Conference and the Scottish National Planning Forum (NPF) guidelines after bariatric multidisciplinary team (MDT) review, were offered surgery. We sought to determine whether target PrWL impacted significantly on PoWL. Our study observed no strong correlation between PrWL and PoWL amongst the patients that had LSG at one and two-three years postoperative years.

In a meta-analysis done in 2011 involving 17 trials and 10 studies by Cassie et al. and a most recent one in 2014 by Gerber et al. (included 23 publications and two review articles), the authors were inconclusive about the effect of PrWL as a result of the heterogeneity in the various study designs [3,9].

In a single-center review of 192 patients who underwent LSG during a nine-month study period, Parmar et al. reported no correlation between those who lost ‘high’ or ‘low’ preoperative weight (based on comparison to the median percent PrWL of 5.1%) and postoperative weight at one year [18].

Another study by Sherman et al. in their review of 141 patients also identified that PrWL is not a reliable predictor of PoWL [11]. Watanabe A et al. in their work noted that the extent of PrWL did not contribute to better weight loss during the overall period [15]. Our findings further align with the above studies that target PrWL does not significantly impact on PoWL after LSG.

We recognise the limitation of this study given its single institutional non-randomised review nature, which may raise the possibility of selection bias and may not entirely project the broader bariatric population.

## Conclusions

Our study observed that achieving target PrWL does not significantly impact PoWL as reported in previous studies. Eligible bariatric candidates should not be denied surgery based on target PrWL. We recommend further prospective trials to delineate the impact of PrWL on LSG outcomes.
